# Roles of MASP-1 and MASP-3 in the development of retinal degeneration in a murine model of dry age-related macular degeneration

**DOI:** 10.3389/fimmu.2025.1566018

**Published:** 2025-03-28

**Authors:** Tomoko Omori, Takeshi Machida, Yumi Ishida, Tetsuju Sekiryu, Hideharu Sekine

**Affiliations:** ^1^ Department of Immunology, Fukushima Medical University School of Medicine, Fukushima, Japan; ^2^ Department of Ophthalmology, Fukushima Medical University School of Medicine, Fukushima, Japan

**Keywords:** complement, lectin pathway, alternative pathway, MASP-1, MASP-3, age-related macular degeneration

## Abstract

Complement is activated through the three different pathways, which are the classical (CP), lectin (LP), and alternative pathways (AP). Complement activation functions to eliminate invading pathogens, whereas dysregulation of complement activation can induce inflammatory disorders such as age-related macular degeneration (AMD). In retinal degeneration induced by sodium iodate (NaIO_3_), a murine model of dry AMD (also called atrophic AMD), it has been suggested that the AP and CP are involved in the disease development. On the other hand, the role of the LP in the development of AMD remains unclear. In the current study, we generated murine dry AMD model with NaIO_3_ using mice deficient for mannose-binding lectin-associated serine protease (MASP)-1 and/or MASP-3, which are required for the LP and AP activation, respectively. Wild-type (WT) C57BL/6J mice showed retinal degeneration, including depigmentation and disruption of the retinal pigment epithelium (RPE), atrophy of the photoreceptor layer (PL), and thinning of the outer nuclear layer (ONL) after NaIO_3_ injection. In contrast, those pathological changes after NaIO_3_ injection were significantly attenuated in MASP-1-deficient (MASP-1^-/-^), MASP-3-deficient (MASP-3^-/-^), and MASP-1/3-double deficient (MASP-1/3^-/-^) mice. These results indicate that both MASP-1 and MASP-3 play a role in photoreceptor degeneration in the NaIO_3_-induced murine dry AMD model. In addition, photoreceptor cell death and retinal C3 activation were observed in NaIO_3_-injected WT mice, whereas those pathological changes were significantly attenuated in NaIO_3_-injected MASP-3^-/-^ and MASP-1/3^-/-^ mice. On the other hand, those pathological changes in NaIO_3_-injected MASP-1^-/-^ mice were comparable to those in NaIO_3_-injected WT mice. Taken together, our results indicate that MASP-3 plays a pivotal role in C3 activation in the retina most likely via activation of the AP leading to the development of retinal degeneration in the NaIO_3_-induced murine dry AMD model. Our results also indicate that MASP-1 plays a role in the development of NaIO_3_-induced retinal degeneration in this murine model, although it remains unclear whether its role in the retinal degeneration is through the LP activation.

## 1 Introduction

The complement system is composed of more than 30 proteins and functions in eliminating invading pathogens and apoptotic cells, thereby maintaining host defense and homeostasis (1, 2). Complement activation initiates through three different pathways: the classical (CP), the lectin (LP), and the alternative pathways (AP). The AP is activated at low levels by spontaneous hydrolysis of C3 to C3(H_2_O). Factor B (FB) associates with C3(H_2_O) and is cleaved by an active form factor D (FD), which is produced by mannose-binding lectin-associated serine protease (MASP)-3, to form a fluid-phase C3 convertase C3(H_2_O)Bb (3, 4). C3(H_2_O)Bb then cleaves C3 into C3a and C3b. C3a is an anaphylatoxin, while C3b binds to nearby surface of pathogens and associates with FB, resulting in FB cleavage by active FD followed by generation of surface-bound (i.e., solid-phase) C3 convertase C3bBb. Thus, at C3b-binding sites, C3 is successively activated via the amplification loop. Activation of the LP and CP is initiated by binding of the pathway-specific pattern-recognition molecules (PRMs) to their targets. Activation of the LP is initiated by binding of the LP PRMs, such as mannose-binding lectin (MBL), ficolins (ficolin-1 (M-ficolin), ficolin-2 (L-ficolin), and ficolin-3 (H-ficolin)), collectin-10 (CL-10 or CL-L1), collectin-11 (CL-11 or CL-K1) and a heteromeric complex of CL-10 and CL-11 (CL-LK), to the surface of pathogens or to apoptotic/necrotic cells (5). Following this binding, MASP-1, complexed with the LP PRMs, is self-activated, and then MASP-2, also complexed with the LP PRMs, is activated by active MASP-1. MASP-2 cleaves both C4 and C2 to form the C3 convertase C4b2b, whereas MASP-1 cleaves only C2. Activation of the CP is initiated by binding of C1q, the CP PRM, to antigen-antibody complexes. Following this binding, serine protease C1r, complexed with C1q, is self-activated, and then C1s, also complexed with C1q, is activated by active C1r. Similarly to MASP-2 in the LP, C1s cleaves C4 and C2 to form the C3 convertase C4b2b. In this way, all three pathways converge at C3 activation, connect to C5 activation and lead to formation of membrane attack complex (C5b-9), causing membrane destabilization.

On the other hand, on the host cells, complement activation is tightly regulated by regulatory proteins such as decay-accelerating factor (DAF or CD55), membrane cofactor protein (MCP or CD46), and factor H (FH), a soluble inhibitor of the AP C3 convertases (1, 2). FH binds to C3b and acts as a cofactor for factor I (FI), which cleaves surface-bound C3b into inactive iC3b that cannot form the AP C3 convertase C3bBb. Dysfunction of complement regulatory proteins, due to aging or gene mutations, leads to dysregulation of complement activation, which is associated with many inflammatory diseases, including age-related macular degeneration (AMD) (6).

AMD is one of the causes of visual impairment in the elderly people. In the early stage of AMD, drusen, which are extracellular deposits of waste product, are observed under the retinal pigment epithelium (RPE), and in the later stage, it progresses to neovascular AMD (also called exudative or wet AMD) with choroidal neovascularization (CNV) or dry AMD (also called atrophic AMD) without CNV. Dry AMD is characterized by loss of RPE, photoreceptors, and choriocapillaris in the macular. Although the pathological mechanism of AMD is not yet fully understood, oxidative stress and persistent inflammation play important role in the development of AMD (7). To date, there is accumulating evidence that the complement system plays a significant role in the pathogenesis of AMD. The association between the Y402H gene polymorphism of FH, a major soluble regulator of the AP, and AMD has been reported (8). This polymorphism has been shown to prevent FH from removing cellular debris, resulting in overwhelming complement activation (9–11). Deposition of C3 and FB on the drusen of AMD patients has been reported (12). These reports suggest that activation of the AP is involved in the development of AMD. On the other hand, Murinello et al. observed deposition of IgG and C1q within the choriocapillaris of AMD patients and deposition of C5b-9 within the drusen and around choriocapillaris, suggesting that the CP is also implicated in the development of AMD (13).

It has also been suggested that the AP and CP are involved in the development of dry AMD-like disease in mice induced by intravenous injection of the oxidizing agent sodium iodate (NaIO_3_). In this model, RPE cells are damaged first, followed by damage to photoreceptor cells maintained by RPE cells, and C1q, C3 and C4 deposition was observed on the damaged photoreceptor outer segments (14). Furthermore, administration of anti-FD antibody into C4-deficient mice protected against NaIO_3_-induced retinal degeneration, suggesting that combined blockade of the CP/LP and AP is required to optimally prevent dry AMD-like disease in mice.

The involvement of the LP in the development of AMD remains largely unclear. In the isolated samples from human donor eyes including RPE, choroid, and neural retina, gene expression levels of LP components were very low as determined by real-time quantitative polymerase chain reaction (RT-qPCR) (15, 16). However, compared to normal donors, increased gene expression levels of ficolin-1 (*FCN1*) in the macular retina of late AMD donors and ficolin-3 (*FCN3*) in the choroidal endothelial cells of early AMD donors have been reported (16). CL-11 was also detected in human retina by RT-PCR (17) and in the neural retina and RPE cells of postmortem human eyes by immunohistochemical staining (18).

We recently reported the levels of C3 and C4 and their activation products C3a and C4a in the aqueous humor of patients with neovascular AMD or cataract (controls) (19). We found that the aqueous humor levels of C3 and C4 were significantly lower in the neovascular AMD eyes compared to the controls. In contrast, the aqueous humor levels of C3a and C4a, as well as the C3a/C3 and C4a/C4 ratios, were significantly higher in the neovascular AMD eyes compared to the controls. We also found that the aqueous humor levels of MASP-2 were significantly lower in the neovascular AMD eyes compared to the controls, possibly due to its consumption. These results suggest that the CP and/or LP are implicated in the development of AMD.

MASP-1 and MASP-3 are splice variants transcribed from the common *Masp1* gene and have identical heavy chains but distinct serine protease domains. We recently generated mice mono-specifically deficient for MASP-1 or MASP-3 by genome editing of the *Masp1* gene using the CRISPR/Cas9 system (4). We found that sera of MASP-1-deficient mice lacked LP activity, whereas sera of MASP-3-deficient mice lacked AP activity. Thus, MASP-1 and MASP-3 are essential complement factors for the physiological activation of the LP and AP, respectively. To clarify whether the LP is involved in the pathogenesis of dry AMD and further investigate the involvement of the AP, we performed histopathological analysis of the retina of a NaIO_3_-induced murine dry AMD model lacking MASP-1 and/or MASP-3.

## 2 Materials and methods

### 2.1 Animals

C57BL/6J mice deficient for MASP-1 (MASP-1^-/-^), MASP-3 (MASP-3^-/-^) (4), or both (MASP-1/3^-/-^) (20) were bred in-house for use in the current study. Wild-type (WT) littermates obtained by mating were used. All experiments were performed using 7- to 12-week-old male mice. All experiments were performed in accordance with the Association for Research in Vision and Ophthalmology (ARVO) Statement and were approved by the Institutional Animal Care and Use Committee Fukushima Medical University (approval no. 2019069, 2021048 and 2023006).

### 2.2 Sodium iodate-induced retinal degeneration

The NaIO_3_ (Sigma-Aldrich, St. Louis, MO, USA) was dissolved in phosphate-buffered saline (PBS), and the mice were injected with 20 mg/kg of NaIO_3_ through a tail vein. For the non-induced control group, WT mice were injected with the same volume of PBS.

### 2.3 Histological analyses

Histological analyses were performed on retinal cross sections stained with hematoxylin and eosin (H&E). After cervical spine dislocation under anesthetized by isoflurane, the eyes were enucleated and immersed in a fixative solution containing superfix (Kurabo, Osaka, Japan) for at least 2 h at room temperature. Paraffin-embedded sections of 5 µm cut through the optic disc of each eye were processed standard manner and stained with H&E. Images of the stained sections were taken with a whole slide scanner (NanoZoomer-SQ; Hamamatsu Photonics, Hamamatsu, Japan) and an Image viewing software (NDP. view2; Hamamatsu Photonics).

### 2.4 RPE flat mount

After removing the excess tissue, the enucleated eye was fixed in 4% paraformaldehyde (PFA) for 20 min on ice, and then washed three times with PBS. The cornea, lens and retina were removed from the eye ball, and four radial cuts were made on the isolated eyecups. The RPE-eyecups were flat mounted and then blocked with Blocking one histo (Nacalai tesque, Kyoto, Japan) containing 0.3% Triron X-100 for 1 h at room temperature for immunofluorochemistry. They were then incubated overnight at 4˚C with a rabbit anti-zonula occludens-1 (ZO-1) polyclonal primary antibody (1:100; Proteintech, Rosemont, IL, USA). After washing the RPE flat mounts, they were incubated for 1 h with an Alexa Flour 488-conjugated donkey anti-rabbit IgG secondary antibody (1:250; BioLegend, San Diego, CA, USA). The RPE flat mounts were washed and mounted in Fluoro-keeper antifade reagent non-hardening type with DAPI (Nacalai tesque) and examined with a microscope (BZ-9000; Keyence, Osaka, Japan). Images of the entire flat mounts were acquired at low magnification, and images of the central, middle, and periphery of the flat mounts were acquired at high magnification. Using an Image J software (version 1.53k; NIH, Bethesda, MD, USA), the percentage of necrotic RPE area, in which the cell shape is unrecognizable, to the total RPE area was calculated.

### 2.5 TUNEL staining

TUNEL staining was performed using *In Situ* Cell Death Detection kit (Roche Biochemicals, Mannheim, Germany) to detect apoptotic cells, according to the manufacturer’s instructions. Eyes enucleated were fixed in 4% PFA for 24 h at 4˚C. The eyes were then immersed in 25% sucrose for at least 48 h at 4˚C, then embedded in optical cutting temperature (OCT) compound (Sakura Finetechnical Co., Ltd., Tokyo, Japan) and snap-frozen in liquid nitrogen. Cryosections of 10 µm thickness cut on glass slides (FRONTIER COAT; Matsunami Glass Ind, Osaka, Japan) were dried at room temperature and then stored at -80˚C until use. After thawing at room temperature, sections were rinsed in PBS and incubated in 0.1% sodium citrate solution containing 0.1% Triton X-100 for 2 min on ice. They were then placed in the TUNEL reaction mixture at 37˚C for 1 h. After washing with PBS, sections were mounted in Fluoro-keeper antifade reagent non-hardening type with DAPI (Nacalai tesque) and photographed using the fluorescent microscope (Nikon, Tokyo, Japan). The number of TUNEL-positive cells in the retinal outer nuclear layer (ONL) was counted within a compartment 400–700 µm from the optic disc.

### 2.6 Immunofluorochemistry

Retinal sections fixed in PFA were thawed at room temperature and rinsed in PBS. After blocked with blocking reagent containing 10% normal goat serum (Nichirei Biosciences, Tokyo, Japan) in PBS containing 0.3% Triton X-100, sections were incubated overnight with FITC-conjugated goat anti-mouse C3 polyclonal antibody (1:100; MP Biomedicals, Santa Ana, CA, USA). Stained sections were then incubated with TrueBlack (Biotium, Fremont, CA, USA) according to the manufacturer’s instructions to quench lipofuscin autofluorescence. The sections were mounted in Fluoro-keeper antifade reagent non-hardening type with DAPI (Nacalai tesque) and photographed using the fluorescent microscope (Nikon). Mean fluorescence intensity (MFI) of C3 staining in the photoreceptor layer (PL), including photoreceptor inner and outer segments, was obtained by measuring the integrated density and area of 500–700 µm from the optic disc using Image J software (NIH). Background fluorescence intensity was subtracted by the average of three readings in areas of the slide that had no tissue.

For MBL-A and C4 staining of mouse retina sections, eyes were enucleated and embedded in OCT compound and frozen in liquid nitrogen. Cryosections of 10 µm thickness were cut, placed on glass slides, dried at room temperature, fixed with cold acetone, and stored at -80˚C until use. Frozen sections were thawed at room temperature before staining. The retinal sections were blocked using 3% Bovine serum albumin (Sigma-Aldrich) in PBS or 10% normal goat serum (Sigma-Aldrich) in PBS containing 0.3% Triton X-100 and then incubated with each primary antibody for MBL-A (1:100; Hycult Biotech, Wayne, PA, USA) and C4 (1:250; Hycult Biotech) overnight at 4˚C. After washing, sections were incubated in a secondary antibody for 1 h at room temperature with FITC-conjugated goat anti-rat IgG (1:500; Beckman Coulter, Brea, CA, USA). Stained sections were mounted in Fluoro-keeper antifade reagent non-hardening type with DAPI (Nacalai tesque) and photographed using the fluorescent microscope (Nikon). MFI of MBL-A or C4 staining in the PL was obtained by measuring the integrated density and area of the presented image using Image J software (NIH). Background fluorescence intensity was subtracted by the average of three readings in areas of the slide that had no tissue.

### 2.7 Western blot analysis

Protein extraction from the retinal tissue was carried out using MT-cell buffer (Sigma Aldrich) supplemented with protease inhibitor cocktail set V (Fujifilm Wako Pure Chemical Corporation, Osaka, Japan). Protein samples were subjected to SDS-PAGE under reducing conditions and electroblotted onto a PVDF membrane. The membrane was blocked with Blocking One (Nacalai tesque) and then incubated with horseradish peroxidase (HRP)-conjugated anti-mouse C3 polyclonal antibody (MP Biomedicals, LLC-Cappel products, Santa Ana, CA, USA) for 1 h at room temperature. Detection was performed using an ECL Prime Western Blotting Detection Reagent (Merck, Darmstadt, Germany) according to the manufacturer’s instructions. Objective protein bands were visualized by chemiluminescence detection using an Amersham Imager 600 (GE healthcare, Buckinghamshire, UK), and band intensities of C3 and iC3b were obtained using an ImageQuant TL software (GE healthcare). After detection of C3 and iC3b, the membranes were incubated with stripping solution (Fujifilm Wako Pure Chemical Corporation) and then subjected to detection of glyceraldehyde 3-phosphate dehydrogenase (GAPDH) using rabbit anti-human GAPDH polyclonal primary antibody (1:5000; GeneTex, Irvine, CA, USA) and HRP-conjugated anti-rabbit IgG secondary antibody (Agilent Technologies, Palo Alto, CA, USA). The band intensity of iC3b and C3 was divided by the band intensity of GAPDH to normalize the total protein amount between samples.

### 2.8 Statistical analyses

Statistical analyses were performed using GraphPad Prism 8 software (GraphPad Software, San Diego, CA, USA). Data are expressed as means and standard error of the mean (SEM). Significance of differences in values was determined by Dunnett’s multiple comparisons test. A P value < 0.05 was considered statistically significant.

## 3 Results

### 3.1 Assessment of retinal degeneration in MASP-1- and/or MASP-3-deficient mice 7 d after NaIO_3_ injection

**Figure 1:**
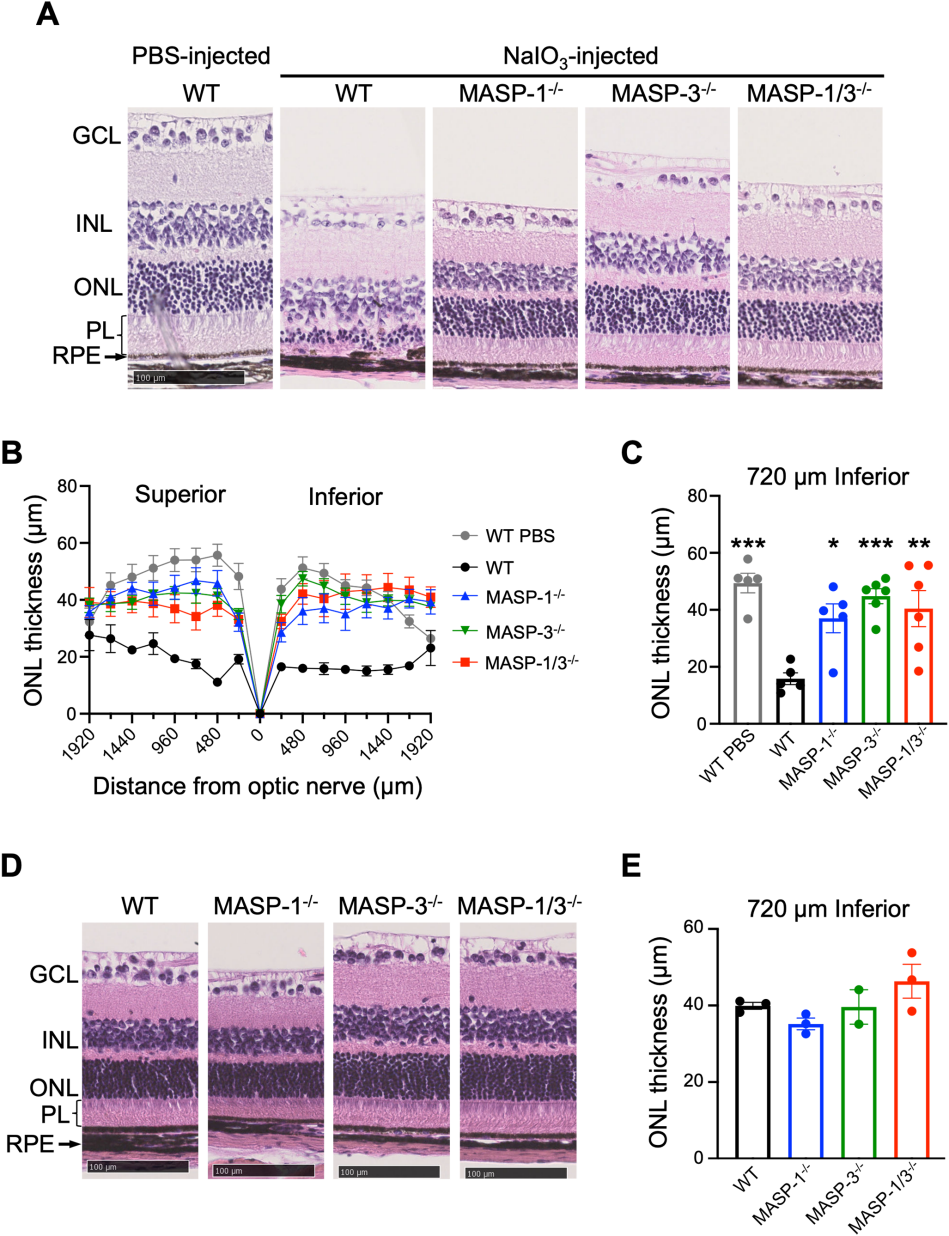
Assessment of retinal degeneration in MASP-1 and/or MASP-3 deficient mice 7 d after NaIO_3_ injection. **(A)** Representative images of H&E-stained retinal sections from PBS-injected WT mice and WT, MASP-1^-/-^, MASP-3^-/-^, and MASP-1/3^-/-^ mice 7 d after NaIO_3_ injection. Scale bars represent 100 µm. **(B)** ONL thickness measured in horizontal sections along superior-inferior axis of the retina. **(C)** ONL thickness at 720 µm inferior to optic nerve of the retina. Error bars indicate SEM of the mean (n = 5–6). Statistical comparisons were performed using Dunnett’s multiple comparisons test (^*^P < 0.05, ^**^P < 0.01, or ^***^P < 0.001 versus NaIO_3_-injected WT group). **(D)** Representative images of H&E-stained retinal sections from WT, MASP-1^-/-^, MASP-3^-/-^, and MASP-1/3^-/-^ mice that were not injected with NaIO_3_. Scale bars represent 100 µm. **(E)** ONL thickness at 720 µm inferior to optic nerve of the retina in WT, MASP-1^-/-^, MASP-3^-/-^, and MASP-1/3^-/-^ mice that were not injected with NaIO_3_. Error bars indicate SEM of the mean (n = 2–3). Dunnett’s multiple comparisons test showed no statistically significant differences between each deficient mice and WT mice. GCL, ganglion cell layer; INL, inner nuclear layer; ONL, outer nuclear layer; RPE, retinal pigment epithelium; PL, photoreceptor layer.

To determine the roles of MASP-1 and/or MASP-3 in NaIO_3_-induced retinal degeneration, morphological change of the RPE and retina was assessed by H&E staining using retinal cross sections of WT, MASP-1^-/-^, MASP-3^-/-^, and MASP-1/3^-/-^ mice 7 d after intravenous NaIO_3_ injection. As shown in **Figure 1A**, the retina of WT mice showed depigmentation and disruption of the RPE and the RPE layer was poorly distinguishable. The inner and outer photoreceptor segments that constitute the PL were absent in the retina of WT mice, and their PL was atrophied with no measurable thickness. Furthermore, the ONL in the retina of WT mice was significantly thinned (**Figures 1B, C**). In contrast, those degenerations and the thinning of ONL were significantly attenuated in MASP-1^-/-^ (P < 0.05), MASP-3^-/-^ (P < 0.001), and MASP-1/3^-/-^ mice (P < 0.01) compared to WT mice (**Figure 1**). To determine whether the observed ONL thinning is dependent on NaIO_3_ injection rather than genetic deficiency of MASP-1 and/or MASP-3, we analyzed the differences in ONL thickness of those deficient mice that were not injected with NaIO_3_. There were no significant differences in ONL thickness between the groups (**Figures 1D, E**).

**Figure 2:**
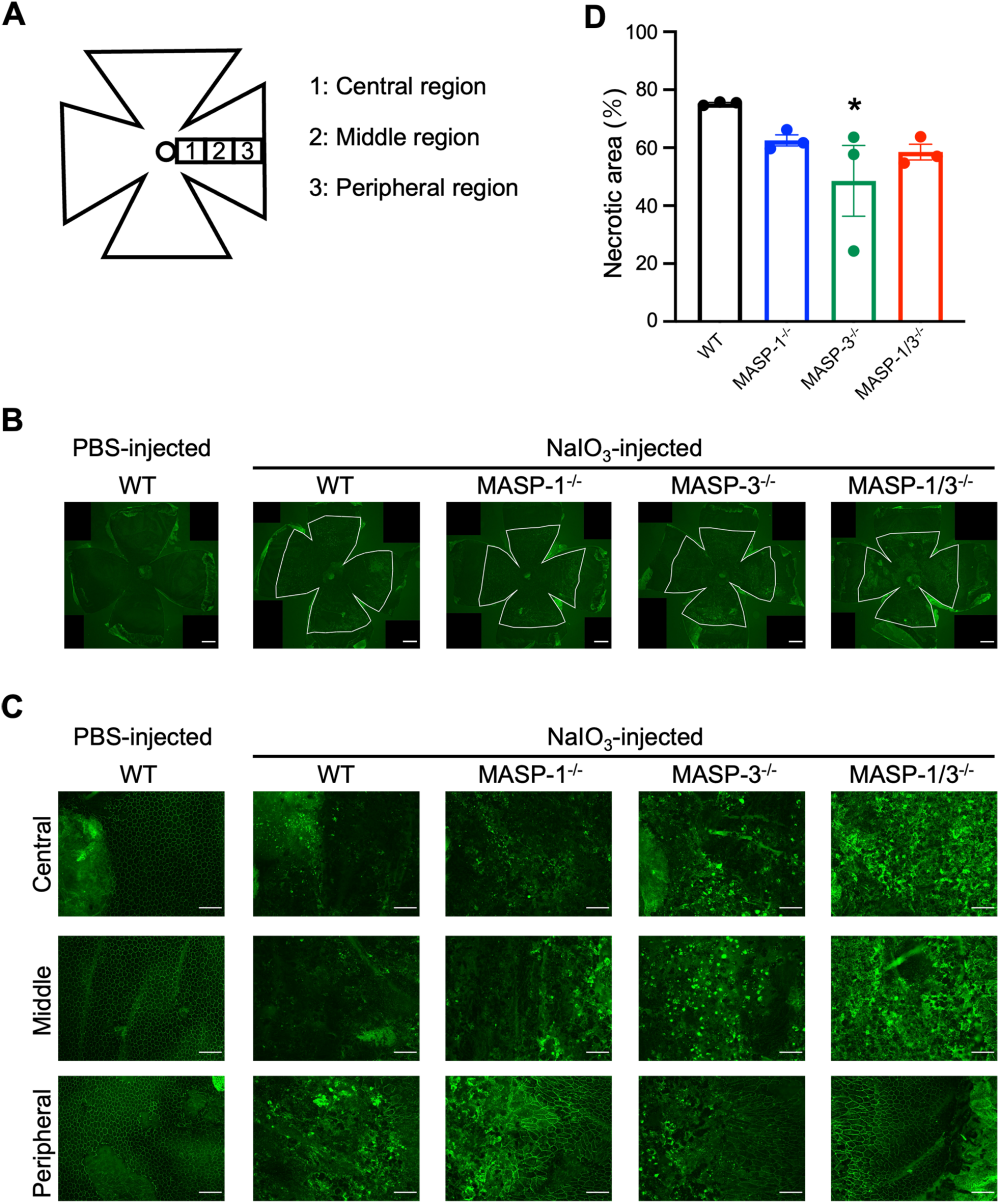
Assessment of morphological damage of RPE in MASP-1 and/or MASP-3 deficient mice 7 d after NaIO_3_ injection. **(A)** RPE flat mounts were used to assess the central, middle, and peripheral regions of the retina. Central region is near the optic nerve. Middle and peripheral regions are 700 µm and 1400 µm from the central region, respectively. **(B)** Representative images of immunofluorescence of ZO-1 (in green) on RPE flat mounts at low magnification. The area of inside white lines shows the necrotic area of RPE flat mounts. Scale bars represent 500 µm. **(C)** Representative images of immunofluorescence of ZO-1 (in green) on RPE flat mounts high magnification images of the central, middle, and peripheral region. Scale bars represent 100 µm. **(D)** To quantify RPE damage, the percentage of necrotic RPE area to total RPE area in each mouse was calculated. Error bars indicate SEM of the mean (n = 3). Statistical comparisons were performed using Dunnett’s multiple comparisons test (^*^P < 0.05 versus NaIO_3_-injected WT group).

To examine the morphological damage of the RPE in the horizontal direction, the RPE/choroid flat mounted sections were stained with antibody against ZO-1, a component protein of the tight junctions between RPE cells. The results showed that RPE cells were severely damaged or lost their normal cell boundary patterns in the posterior central region in all groups 7 d after NaIO_3_ injection (**Figure 2**). In the peripheral region, RPE cells exhibited elongated or intact RPE in MASP-1^-/-^, MASP-3^-/-^, and MASP-1/3^-/-^ mice, whereas were damaged in WT mice (**Figure 2C**). Necrotic area of RPE flat mounts, illustrated as areas within white lines in **Figure 2B**, immunostained with anti-ZO-1 antibody was quantified using Image J software and expressed as a percentage. As shown in **Figure 2D**, the percentage of necrotic RPE area to total RPE area 7 d after NaIO_3_ injection was 75.30 ± 0.36% in WT mice, 62.53 ± 1.92% in MASP-1^-/-^ mice (not significantly decreased compared to WT mice), 48.57 ± 12.20% in MASP-3^-/-^ mice (significantly decreased at P < 0.05 compared to WT mice), and 58.50 ± 2.73% in MASP-1/3^-/-^ mice (not significantly decreased compared to WT mice). Only MASP-3^-/-^ mice showed statistically significant decrease in the percentage of necrotic RPE area compared to WT mice, however, both MASP-1^-/-^ and MASP-1/3^-/-^ mice also showed a trend toward smaller necrotic RPE area than WT mice 7 d after NaIO_3_ injection. These results suggest that MASP-1 and MASP-3 play a role in photoreceptor degeneration in the NaIO_3_-induced murine dry AMD model.

### 3.2 Changes in the number of apoptotic photoreceptor cells in MASP-1 and/or MASP-3 deficient mice after NaIO_3_ injection

**Figure 3:**
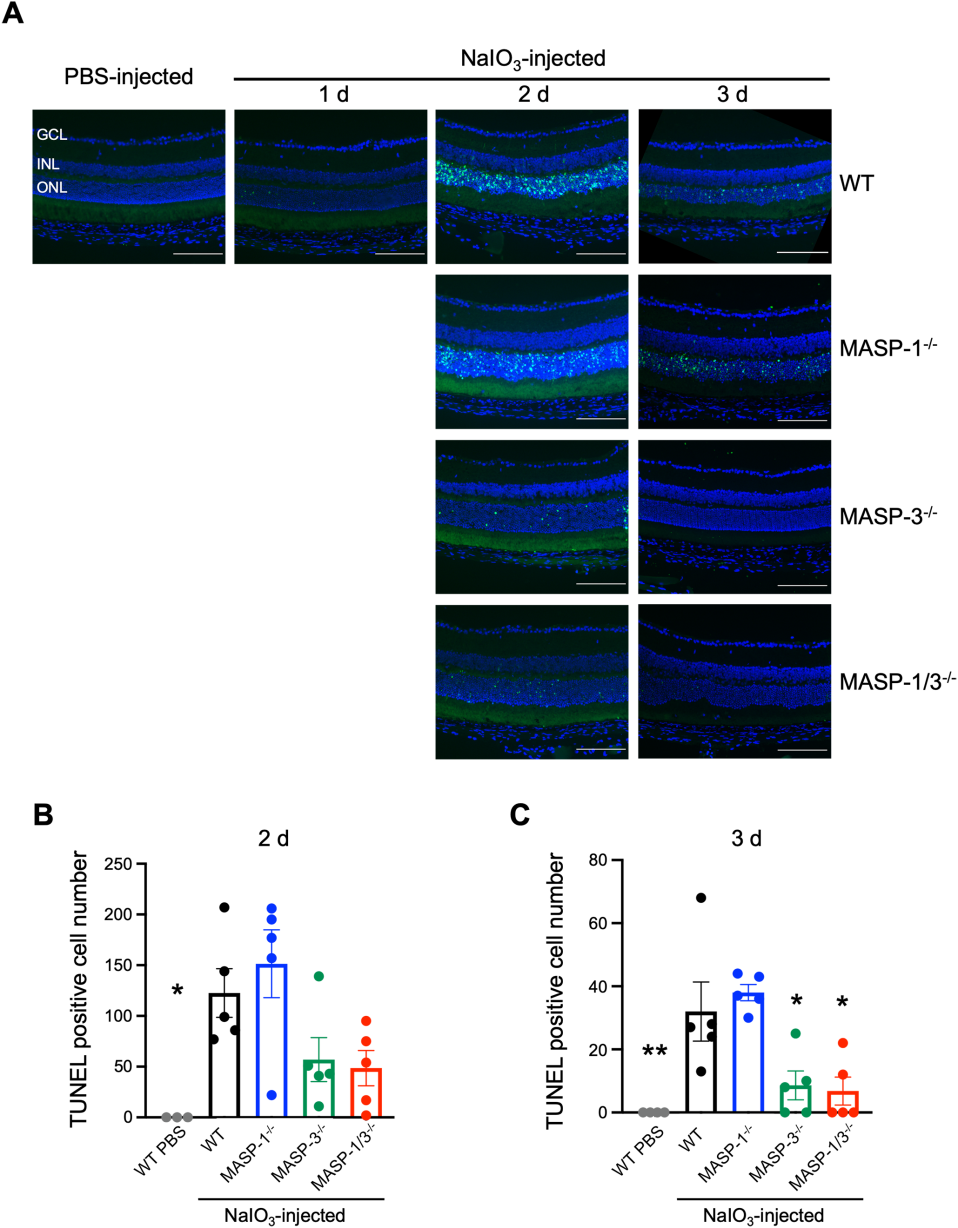
Changes in the number of apoptotic photoreceptor cells in MASP-1 and/or MASP-3 deficient mice after NaIO_3_ injection. **(A)** Representative images of TUNEL (in green) and DAPI (in blue) stained retinal sections showing the entire compartment 400–700 μm from the optic disc. Scale bars represent 100 µm. Retinal sections of WT mice on days 1, 2, and 3 after NaIO_3_ injection and those of MASP-1^-/-^, MASP-3^-/-^, and MASP-1/3^-/-^ mice on days 2 and 3 after NaIO_3_ injection. **(B, C)** Quantitative analysis of the number of TUNEL positive cells in ONL on days 2 **(B)** and 3 **(C)** after NaIO_3_ injection, counted within a compartment 400–700 µm from the optic disc. Error bars indicate SEM of the mean (n = 3–5). Statistical comparisons were performed using Dunnett’s multiple comparisons test (^*^P < 0.05 and ^**^P < 0.01 versus NaIO_3_-injected WT group). GCL, ganglion cell layer; INL, inner nuclear layer; ONL, outer nuclear layer.

To evaluate the effect of MASP-1 and/or MASP-3 deficiency on photoreceptor degeneration, TUNEL staining was performed on cryosections of enucleated mouse eyes. TUNEL-positive cells in the ONL of WT mice were almost absent on day 1 and then increased on day 2 and 3 after NaIO_3_ injection (**Figure 3A**). Comparisons on day 2 after NaIO_3_ injection showed a trend toward fewer TUNEL-positive cells in the ONL of MASP-3^-/-^ (P = 0.19) and MASP-1/3^-/-^ mice (P = 0.12) compared with WT mice (**Figures 3A, B**). Comparison on day 3 after NaIO_3_ injection showed statistically significantly fewer TUNEL-positive cells in the ONL of MASP-3^-/-^ (P = 0.02) and MASP-1/3^-/-^ mice (P = 0.01) compared with WT mice (**Figures 3A, C**). On the other hand, there were no statistically significant differences in the number of TUNEL-positive cells in the ONL between WT and MASP-1^-/-^ mice on either day 2 or 3 after NaIO_3_ injection (**Figures 3B, C**). These results suggest that the absence of MASP-3, but not MASP-1, prevented apoptotic photoreceptor cell death in the NaIO_3_-induced murine dry AMD model.

### 3.3 Assessment of C3 deposition and activation in retina of MASP-1 and/or MASP-3 deficient mice 2 d after NaIO_3_ injection

**Figure 4:**
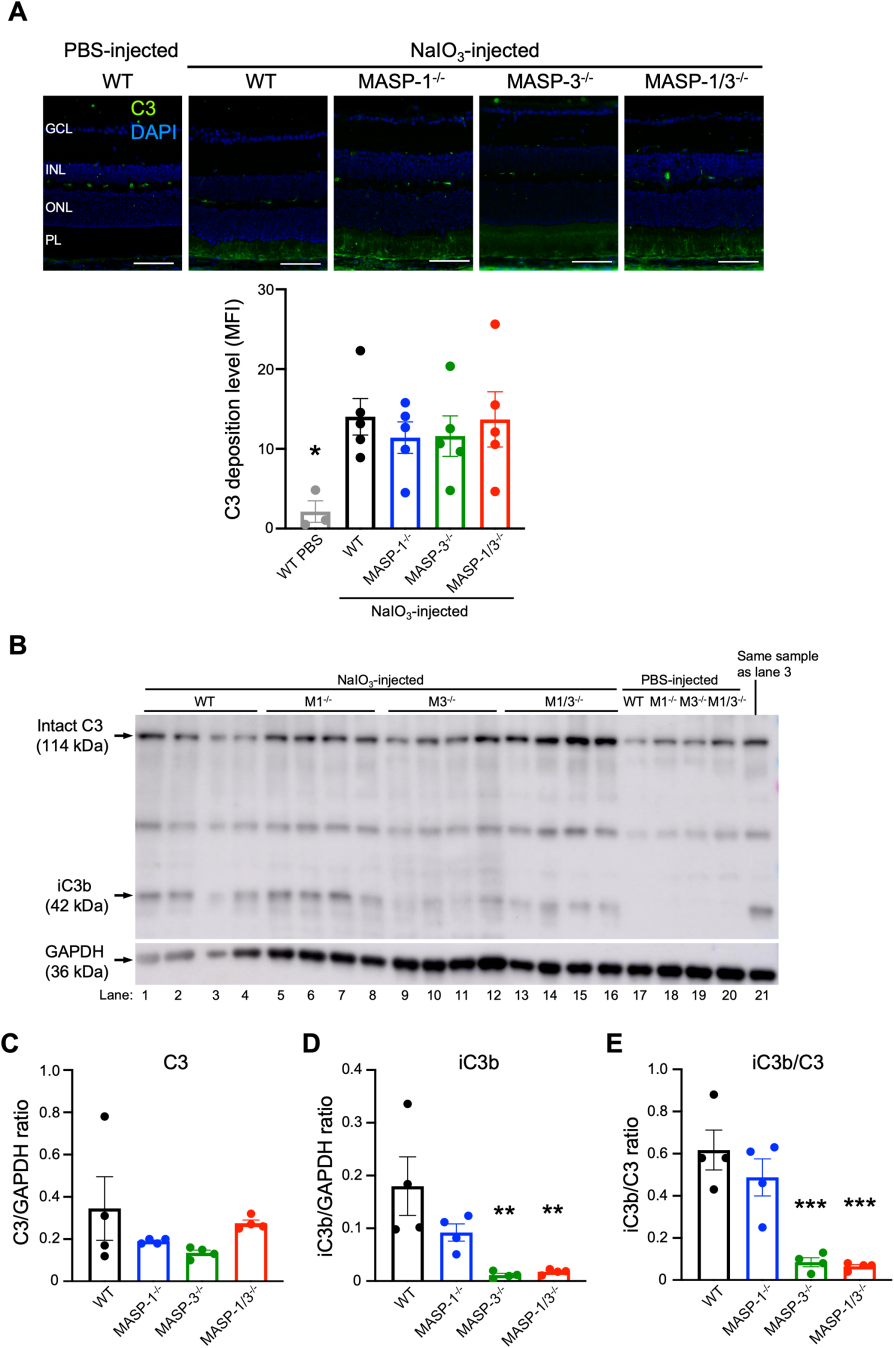
Assessment of C3 deposition and activation levels in retina of MASP-1 and/or MASP-3 deficient mice 2 d after NaIO_3_ injection. **(A)** Representative images of C3 (in green) and DAPI (in blue) stained retinal sections from PBS-injected WT mice and WT, MASP-1^-/-^, MASP-3^-/-^, and MASP-1/3^-/-^ mice 2 d after NaIO_3_ injection. Scale bars represent 50 µm. The bar graph shows mean fluorescence intensity (MFI) of C3 staining in the photoreceptor layer (PL) of each mouse. Error bars indicate SEM of the mean (n = 3–5). Statistical comparisons were performed using Dunnett’s multiple comparisons test (*P < 0.05 versus NaIO_3_-injected WT mice group). **(B)** Western blot of C3 protein in the neural retina from WT, MASP-1^-/-^ (M1^-/-^), MASP-3^-/-^ (M3^-/-^), and MASP-1/3^-/-^ (M1/3^-/-^) mice 2 d after NaIO_3_ or PBS injection. Lane 3 was excluded from the quantitative analysis due to an error in sample addition, and lane 21, which is the same sample as lane 3, was used for the quantitative analysis. **(C–E)** Quantitative analysis of western blots for C3 **(C)** or iC3b **(D)** and the iC3b/C3 ratio **(E)** after normalization with GAPDH. Error bars indicate SEM of the mean (n = 4). Statistical comparisons were performed using Dunnett’s multiple comparisons test (^**^P < 0.01 and ^***^P < 0.001 versus NaIO_3_-injected WT group). GCL, ganglion cell layer; INL, inner nuclear layer; ONL, outer nuclear layer; PL, photoreceptor layer.

To assess the extent of complement activation in retinal degeneration, immunofluorescence staining of C3 was performed on the retinal tissue of mice 2 d after NaIO_3_ injection. As shown in **Figure 4A**, C3 was detected in the PL of WT, MASP-1^-/-^, MASP-3^-/-^, and MASP-1/3^-/-^ mice 2 d after NaIO_3_ injection. There was no statistically significant difference in the fluorescence intensity of C3 in the PL between the groups (**Figure 4A**). On the other hand, C3 was little-to-no detected in the ONL of all groups 2 d after NaIO_3_ injection, and there was no significant difference in the fluorescence intensity of C3 in the PL between the groups (**Figure 4A**).

Since the anti-mouse C3 polyclonal antibody used in this study can detect both intact C3 and its activation products (e.g., C3b, iC3b, C3d, and C3dg), it is possible that the C3 detected in the PL of mice 2 d after NaIO_3_ injection may not reflect *in situ* C3 activation. To address the issue, we performed Western blot analysis of C3 molecules in the protein lysates of neural retinas isolated from WT, MASP-1^-/-^, MASP-3^-/-^, or MASP-1/3^-/-^ mice 2 d after NaIO_3_ injection. As shown in **Figure 4B**, intact C3 α-chain was detected in the lysates from all groups including the PBS-injected group. On the other hand, iC3b was not detected in the lysates from the PBS-injected group, but was detected in those from all groups 2 d after NaIO_3_ injection. Comparison of the data on the band intensities of iC3b and C3 normalized with that of GAPDH for each mouse revealed that there was no statistically significant difference in intact C3 level between the groups (**Figure 4C**). Notably, iC3b levels in the lysates from MASP-3^-/-^ (P < 0.01) and MASP-1/3^-/-^ mice (P < 0.01) were significantly lower than those from WT mice (**Figure 4D**). There was a trend toward lower iC3b levels in the lysates from MASP-1^-/-^ mice compared to those from WT mice, although the difference between MASP-1^-/-^ and WT mice did not reach statistical significance (P = 0.129, **Figure 4D**). To evaluate the degree of intraocular C3 activation in each mouse, the iC3b/C3 ratio was calculated using these band intensities. Consistent with the result of iC3b levels, the iC3b/C3 ratios in the lysates from MASP-3^-/-^ (P < 0.001) and MASP-1/3^-/-^ (P < 0.001) mice were significantly lower than those from WT mice (**Figure 4E**). The iC3b/C3 ratio in lysates from MASP-1^-/-^ mice also showed lower levels compared with those from WT mice, although the difference between MASP-1^-/-^ and WT mice did not reach statistical significance (P = 0.394). Thus, a significant reduction in C3 activation levels was observed in mouse retinas 2 d after NaIO_3_ injection in the absence of MASP-3, but not in the absence of MASP-1.

### 3.4 Changes over time in C4 and MBL deposition in photoreceptor layer of mice after NaIO_3_ injection

**Figure 5:**
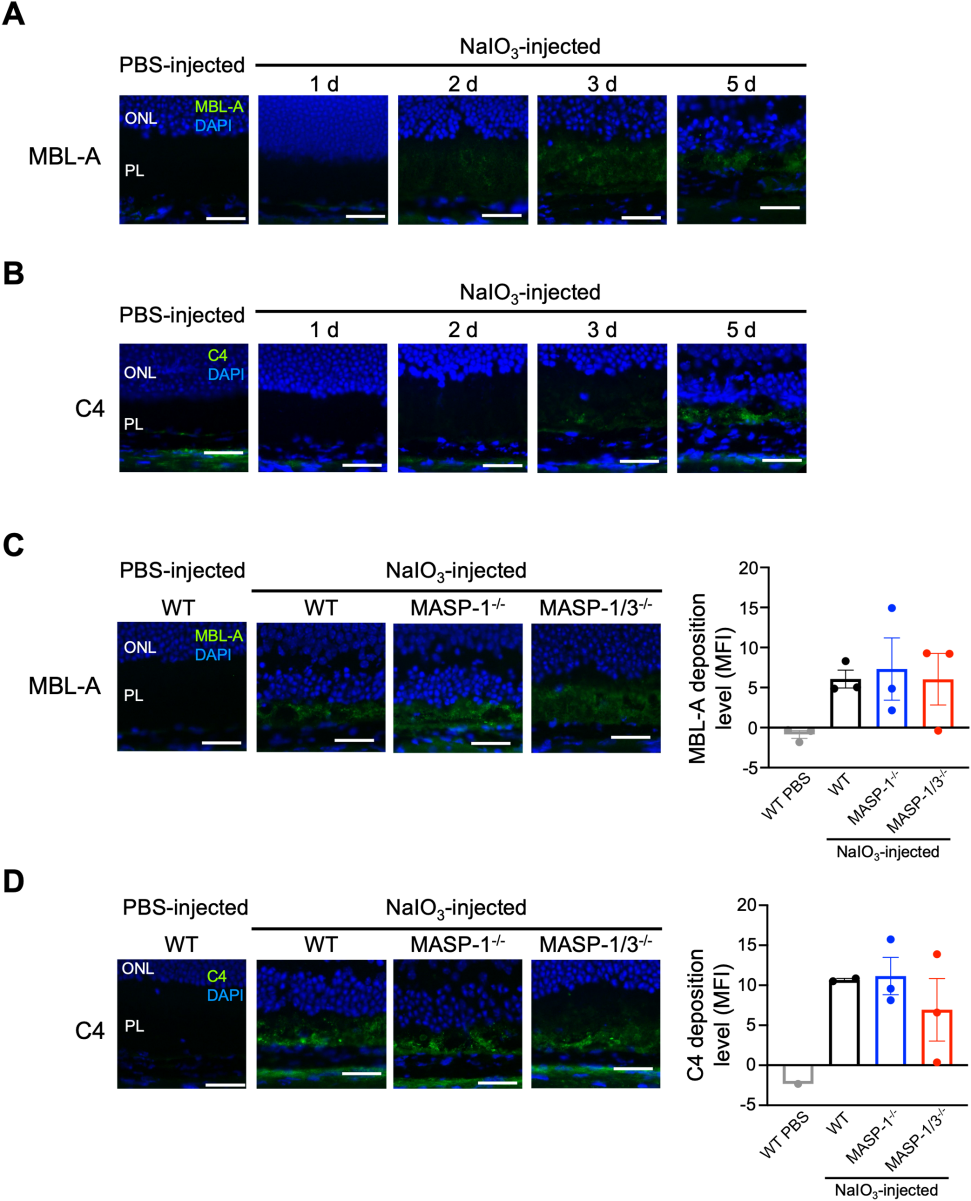
Changes in C4 and MBL-A deposition in photoreceptor layer of MASP-1^-/-^ and MASP-1/3^-/-^ mice after NaIO_3_ injection. Representative images of MBL-A (in green) and DAPI (in blue) **(A)** or C4 (in green) and DAPI (in blue) **(B)** stained retinal sections from PBS-injected WT mice (7 d after injection) and WT mice on days 1, 2, 3, and 5 after NaIO_3_ injection (n = 3 for **A** and n = 1 for **B**). Representative images of MBL-A (in green) and DAPI (in blue) **(C)** stained retinal sections from PBS-injected WT mice (7 d after injection, n = 3) and WT (n = 3), MASP-1^-/-^ (n = 3), and MASP-1/3^-/-^ (n = 3) mice on day 5 after NaIO_3_ injection, or C4 (in green) and DAPI (in blue) **(D)** stained retinal sections from PBS-injected WT mice (2 d after injection, n = 1) and WT (n = 2), MASP-1^-/-^ (n = 3), and MASP-1/3^-/-^ (n = 3) mice on day 5 after NaIO_3_ injection. Scale bars represent 20 µm. The bar graph shows MFI of MBL-A **(C)** or C4 **(D)** staining in the photoreceptor layer (PL) of each mouse. Error bars indicate SEM. Statistical comparisons were performed using Dunnett’s multiple comparisons test (versus NaIO_3_-injected WT mice group). ONL, outer nuclear layer; PL, photoreceptor layer.

In the current study, we found that both MASP-1 and MASP-3 play critical roles in photoreceptor degeneration in the NaIO_3_-induced murine dry AMD model. However, unlike MASP-3-deficient mice, no significant reduction in C3 activation levels was observed in the retina of MASP-1-deficient mice 2 d after NaIO_3_ injection. To determine whether LP activation, in which MASP-1 plays a key role, is involved in the reduction of retinal degeneration, deposition of MBL-A and C4 in the retina of mice on days 1, 2, 3, and 5 after NaIO_3_ injection was evaluated by immunofluorescence staining. As shown in **Figure 5A**, MBL-A was detected in the PL of WT mice on days 2, 3, and 5 after NaIO_3_ injection. Whereas C4 was detected in the PL of WT mice on day 3 and 5 after NaIO_3_ injection. Based on these observations, we assessed the levels of MBL and C4 deposition in the retinas of MASP-1^-/-^ and MASP-1/3^-/-^ mice on day 5 after NaIO_3_ injection. As shown in **Figures 5C, D**, deposition of MBL-A and C4 was also observed in the PL of MASP-1^-/-^ and MASP-1/3^-/-^ mice, similar to that in WT mice. There were no significant differences in MBL-A and C4 deposition levels in the PL between the three groups. These results suggest that the absence of MASP-1 did not affect the levels of C4 deposition in the retinas of those mice 5 d after NaIO_3_ injection.

## 4 Discussion

To clarify whether the LP is involved in the pathogenesis of dry AMD and to further investigate the involvement of the AP, we performed histopathological analysis of the retina of a NaIO_3_-induced murine dry AMD model using mice lacking MASP-1 and/or MASP-3. NaIO_3_ is oxidative stress inducer and selectively damages RPE cell by increasing the ability of melanin to convert glycine to glyoxylate, which inhibits the enzymatic activity of intracellular energy generating processes (21). NaIO_3_ also induces the production of reactive oxygen species (ROS), which injure mitochondria in RPE cells (22). The results presented in this report indicate that the absence of MASP-1 or MASP-3 in a murine dry AMD model has a significantly beneficial effect on the development of the NaIO_3_-induced retinal degeneration. Mice lacking MASP-1, MASP-3, or MASP-1/3 showed significantly reduced RPE depigmentation and destruction, PL atrophy, and ONL thinning after NaIO_3_ injection compared with WT mice. Mice lacking MASP-3 or MASP-1/3 also showed significantly decreased numbers of TUNEL-positive apoptotic cells in the ONL and retinal C3 activation levels after NaIO_3_ injection compared with WT mice. In contrast, these beneficial effects were not observed in mice lacking MASP-1 alone.

The first studies suggesting a link between AMD and complement can be traced back to reports showing the deposition of various complement proteins along drusen and Bruch’s membrane in patients with AMD (23). Subsequently, it was reported that genetic polymorphisms in FH, which selectively regulates the AP, are risk factors for both dry and neovascular AMD (8, 24–27). In addition, polymorphisms in the genes encoding FB and C3 of the AP, as well as in the FH-like gene, have been reported to be associated with AMD (28–30). A strong association between AMD and the AP has also been reported in murine models of AMD. Rohrer and colleagues demonstrated that in a laser-induced murine neovascular AMD model, mice lacking either the FD or FB of the AP were protected from the development of CNV compared with WT mice (31, 32). In the present study, we used MASP-3-deficient mice to investigate whether the AP is also involved in the development of retinal degeneration in a NaIO_3_-induced murine dry AMD model. As the result, we observed that mice lacking MASP-3 were protected from the development of retinal degeneration after NaIO_3_ injection compared with WT mice.

MASP-3 is a splice variant transcribed from the *Masp1* gene, which also encodes the LP complement factor MASP-1 and its competitive protein MAP-1 (or MAp44) lacking the enzyme active site (3). We previously reported that in MASP-3-deficient mice, FD circulates in an inactive form, resulting in the loss of serum AP activity (4). This is because the active form of FD can efficiently cleave FB bound to C3(H_2_O) or C3b to generate the AP C3 convertases C3(H_2_O)Bb or C3bBb, respectively (3). C3(H_2_O) is formed in the fluid-phase by spontaneous hydrolysis of the thioester of C3 in a process called “tick-over”. On the other hand, the initial C3b can be formed on the solid-phase by nearby C3(H_2_O)Bb or by the CP/LP C3 convertase C4b2b. Once C3bBb is generated on the solid-phase, it cleaves C3 there, allowing efficient deposition of C3b on the target. The deposited C3b serves as a source of additional C3bBb and ultimately contributes to the amplification loop of the AP. In fact, approximately 80% of the C3 convertase deposited on the target surface originates from the AP amplification loop, rather than from the CP or LP (33, 34). Based on these reports, it is considered that MASP-3 plays an important role in complement activation by participating in the generation of the amplification loop C3 convertase C3bBb, which contributes significantly to C3 activation on the target surface. Thus, the significant decrease in retinal C3 activation levels observed in MASP-3-deficient mice 2 d after NaIO_3_ injection is most likely due to a decrease in the generation of C3bBb in the retina.

Decreased generation of C3bBb in retinal tissue leads to decreased generation of the C3bBb-derived C5 convertase, C3bBb3b, and thus reduced production of the anaphylatoxin C5a and formation of the membrane attack complex C5b-9. The formation of C5b-9 on RPE cells triggers an increase in calcium influx into the cells, leading to mitochondrial damage and an increase in ROS production (35). Thus, complement activation mediates a feed-forward cycle of mitochondrial injury resulting in loss of RPE homeostasis, which is essential for maintaining photoreceptor cells through nutrient transport and outer segment phagocytosis (36). Indeed, MASP-3-deficient mice were protected from TUNEL-positive apoptotic photoreceptor cell death on day 3 after NaIO_3_ injection and from RPE depigmentation and disruption, PL atrophy, and thinning of the ONL on day 7 after NaIO_3_ injection. Furthermore, MASP-3-deficient mice showed a decrease in the percentage of necrotic RPE area 7 d after NaIO_3_ injection, which is consistent with the results of anti-C5 antibody administration in a NaIO_3_-induced murine dry AMD model (14). Those mice administered with anti-C5 antibody showed the loss of central RPE integrity, but prevented the loss of peripheral RPE integrity, suggesting that C5 activation may affect the propagation of RPE cell death into the periphery (14, 37). Taken together, the present study suggested that MASP-3 acts as an exacerbating factor in NaIO_3_-induced RPE cell injury and subsequent photoreceptor cell damage via activation of the AP.

MASP-1 activates MASP-2 and C2, contributing to the generation of the LP C3 convertase C4b2b. In the present study, mice lacking MASP-1 showed significantly attenuated ONL thinning on day 7 after NaIO_3_ injection, similar to mice lacking MASP-3 or MASP-1/3. However, unlike mice lacking MASP-3 or MASP-1/3, mice lacking MASP-1 alone did not show a decrease in the number of TUNEL-positive apoptotic cells in the ONL or in the retinal C3 activation levels after NaIO_3_ injection. These results suggest that the absence of MASP-1 alone has little-to-no effect on the retinal C3 activation levels in the NaIO_3_-induced murine dry AMD model. To clarify whether there was an association between the LP and NaIO_3_-induced retinal degeneration, we performed immunofluorescence staining for C4 and MBL-A in the retinal sections from mice after NaIO_3_ injection. Deposition of MBL-A and C4 was clearly observed in the PL of WT mice 5 d after NaIO_3_ injection, suggesting that the LP is activated in their retinal tissue. Notably, after NaIO_3_ injection, TUNEL-positive cells and C3 deposition were observed before MBL-A and C4 deposition in their retinal tissue. These observations suggest that AP activation occurs first, followed by LP activation in the retina after NaIO_3_ injection. MBL can bind to late apoptotic cells and enhance phagocytosis of apoptotic cells by macrophages (38). However, transcription levels of MBL in the human retina are minimal (15), suggesting that MBL deposits observed in the PL after NaIO_3_ injection are unlikely to be derived from locally produced MBL. In the NaIO_3_-induced murine dry AMD model, it is suggested that the blood-retinal barrier is disrupted, allowing circulating MBL-A to enter the retina and potentially play a role in the removal of apoptotic cells by opsonization. It is unclear whether the deposition of C4 observed in the PL after NaIO_3_ injection is a result of LP activation. C4 can be activated through the CP as well as the LP. Indeed, it has been reported that the CP is involved in the development of NaIO_3_-induced retinal degeneration (14). In the present study, no significant difference was observed in the levels of C4 deposition in the PL between MASP-1-deficient and WT mice, suggesting that the retinal C4 deposition observed 5 d after NaIO_3_ injection was not caused by intraretinal activation of the LP. Further studies are needed to determine whether the LP is involved in the development of retinal degeneration in the NaIO_3_-induced murine dry AMD model and in human dry AMD.

The question remains as to why retinal degeneration was attenuated in mice lacking MASP-1 alone, as in mice lacking MASP-3 or MASP-1/3. Recently, the rate of choriocapillaris flow deficit in participants with large drusen was measured at 6-month intervals, and impaired choriocapillaris blood flow was found prior to the development of nascent geographic atrophy (39). In a rabbit model of NaIO_3_-induced dry AMD, fibrin deposition was observed in the choroidal interstitium and Bruch’s membrane surrounding the narrowed choriocapillaris 3 h after NaIO_3_ injection (40). Furthermore, reduced retinal blood flow has been reported in a NaIO_3_-induced murine dry AMD model (41). These results suggests that the blood coagulation system is involved in the development of retinal degeneration in human dry AMD and in the NaIO_3_-induced models. Besides LP activation, MASP-1 cleaves coagulation factors such as fibrinogen and factor XIII to form fibrin clots, and also cleaves high-molecular-weight kininogen, a member of kallikrein-kinin system, to promote vasorelaxation and vasodilation (42). In fact, it has been reported that thrombin-like activity on mannan-coated plates, to which MBL/MASPs complexes can bind, was markedly reduced in the sera of MASP-1/3^-/-^ mice compared with that of MASP-2^-/-^ or WT mice (43). In the present study, the absence of MASP-1 may have reduced fibrin deposition in the choriocapillaris, which plays an essential role in RPE cell survival, thereby preserving blood flow and alleviating retinal degeneration after NaIO_3_ injection. Interestingly, MASP-1 levels in plasma of patients with neovascular AMD were higher compared with healthy controls and patients with early AMD (44). Elevated levels of circulating MASP-1 may influence the reduction in choroidal blood flow prior to the development of retinal degeneration in AMD through activation of coagulation factors. However, further studies are required to clarify whether the blood coagulation system is involved in the development of retinal degeneration in the NaIO_3_-induced models and in human dry AMD.

In summary, we demonstrated that MASP-1 and MASP-3 act as exacerbating factors in the NaIO_3_-induced murine dry AMD model. MASP-3 is critically involved in apoptosis and C3 activation (i.e., AP activation) in the retina after NaIO_3_ injection, suggesting that MASP-3 could be a therapeutic target for dry AMD. On the other hand, no clear evidence was observed that MASP-1 is involved in retinal degeneration in the NaIO_3_-induced model via complement activation. Further studies are needed to define the role of MASP-1 in the development of retinal degeneration in the NaIO_3_-induced murine dry AMD model.

## Data Availability

The original contributions presented in the study are included in the article/supplementary material. Further inquiries can be directed to the corresponding author.
